# Model-informed precision dosing in vancomycin treatment

**DOI:** 10.3389/fphar.2023.1252757

**Published:** 2023-10-09

**Authors:** Sukyong Yoon, Jinju Guk, Sang-Guk Lee, Dongwoo Chae, Jeong-Ho Kim, Kyungsoo Park

**Affiliations:** ^1^ Department of Pharmacology, Yonsei University College of Medicine, Seoul, Republic of Korea; ^2^ Brain Korea 21 Plus Project for Medical Science, Yonsei University, Seoul, Republic of Korea; ^3^ Department of Laboratory Medicine, Yonsei University College of Medicine, Seoul, Republic of Korea

**Keywords:** vancomycin, pharmacokinetic, pharmacodynamic, NONMEM, C-reactive protein, model-informed precision dosing, optimal dose, R shiny

## Abstract

**Introduction:** While vancomycin remains a widely prescribed antibiotic, it can cause ototoxicity and nephrotoxicity, both of which are concentration-associated. Overtreatment can occur when the treatment lasts for an unnecessarily long time. Using a model-informed precision dosing scheme, this study aims to develop a population pharmacokinetic (PK) and pharmacodynamic (PD) model for vancomycin to determine the optimal dosage regimen and treatment duration in order to avoid drug-induced toxicity.

**Methods:** The data were obtained from electronic medical records of 542 patients, including 40 children, and were analyzed using NONMEM software. For PK, vancomycin concentrations were described with a two-compartment model incorporating allometry scaling.

**Results and discussion:** This revealed that systemic clearance decreased with creatinine and blood urea nitrogen levels, history of diabetes and renal diseases, and further decreased in women. On the other hand, the central volume of distribution increased with age. For PD, C-reactive protein (CRP) plasma concentrations were described by transit compartments and were found to decrease with the presence of pneumonia. Simulations demonstrated that, given the model informed optimal doses, peak and trough concentrations as well as the area under the concentration-time curve remained within the therapeutic range, even at doses smaller than routine doses, for most patients. Additionally, CRP levels decreased more rapidly with the higher dose starting from 10 days after treatment initiation. The developed R Shiny application efficiently visualized the time courses of vancomycin and CRP concentrations, indicating its applicability in designing optimal treatment schemes simply based on visual inspection.

## 1 Introduction

Vancomycin is a glycopeptide antibiotic used to treat infections caused by vancomycin susceptible bacterial species, especially methicillin-resistant *Staphylococcus aureus* (Levine, 2008). It is also indicated for the treatment of infections caused by Gram-positive bacteria in patients with allergies to beta-lactam antibiotics or cephalosporins (Rybak M. et al., 2009). Due to its various indications, vancomycin has been increasingly used in many countries since it was approved (Kirst et al., 1998) and it remains a widely prescribed treatment agent for various bacterial infectious diseases.

Vancomycin, however, can cause severe toxicities such as ototoxicity and nephrotoxicity, which can lead to treatment failure if not carefully used. In past years, it was believed that the toxicity was associated with impurities rather than its concentration (Moellering, 1984). Subsequently, with the introduction of newer manufacturing processes, ototoxicity has almost disappeared and nephrotoxicity has also decreased significantly (Brummett, 1981; Farber and Moellering, 1983).

Regarding nephrotoxicity, antioxidants have shown a nephroprotective effect against vancomycin associated nephrotoxicity. In a study assessing the nephroprotective role of ascorbic acid against vancomycin associated nephrotoxicity, co-administration of ascorbic acid with vancomycin preserved renal function and reduced the absolute risk of nephrotoxicity by 20.3%“ (Hesham El-Sherazy et al., 2021). Investigating the correlation between nephrotoxicity and different antibiotic regimens, a comparison was made between once-daily dosing and individualized multiple daily dosing of gentamicin and amikacin in terms of clinical and bacteriological efficacy. The study assessed the incidence of nephrotoxicity associated with both regimens and found a non-significant difference between the two dosing regimens” (Abdel-Bari et al., 2011).

Contrary to past understanding, it has been found that high doses of vancomycin can also increase toxicity (Hidayat et al., 2006). Recent studies have investigated the relationship between concentration and nephrotoxicity, as well as the potential nephrotoxicity when vancomycin is administered with aminoglycoside antibiotics (Elyasi et al., 2012; Gelfand and Cleveland, 2013).

Vancomycin treatment failure can also occur in bacterial-resistant infections (Centers for Disease Control and Prevention (CDC). Antibiotic Resistance Threats in the United States, 2019. Atlanta, GA: U.S. Department of Health and Human Services, Centers for Disease Control and Prevention, 2019). Since vancomycin-resistant enterococci (VRE) was reported in 1980s, six resistance patterns have been identified (Kim et al., 2000; Murray, 2000; Depardieu et al., 2003) Various strategies to mitigate resistance have been introduced (Chong and Lee, 2000), including the administration of vancomycin to achieve AUC/MIC = 400 (Martin et al., 2010), with AUC denoting the area under the concentration-time curve and MIC denoting the minimum inhibitory concentration, defined as the lowest concentration capable of inhibiting bacterial growth. However, for bacteria strains with relatively high MICs, the AUC/MIC = 400 requirement may be inadequate for effective treatment.

Given that avoiding toxicity and resistance is crucial for the success of vancomycin therapy, efforts have been made to investigate factors affecting drug exposure and to adjust the dose accordingly. Early efforts in this direction include a dosing interval nomogram to achieve target peak and trough concentrations, adjusted based on body weight and renal function (Matzke et al., 1984). Additionally, a consensus review by American academic societies recommended weight-normalized loading and maintenance doses to achieve target trough concentrations or target AUC/MIC ratios (Rybak M. J. et al., 2009).

However, these early descriptions of vancomycin plasma concentrations did not utilize a nonlinear mixed-effects (NLME) population pharmacokinetic (PK) model, which is now a standard model for drug concentrations. This model distinguishes inter-individual variability from intra-individual or residual variability, producing more reliable results in PK analyses and dose selection. Dose optimization based on a NLME population PK model falls under the category of model-informed precision dosing (MIPD). Numerous works have demonstrated the novelty of MIPD in achieving vancomycin dose optimization (Frymoyer et al., 2020; Ter Heine et al., 2020; Hughes et al., 2021; Uster et al., 2021; Aljutayli et al., 2022; Heus et al., 2022; Matsumoto et al., 2022). These works include model averaging and selection algorithms to improve predictive performance (Uster et al., 2021; Heus et al., 2022), the application of published models for prospective validation (Ter Heine et al., 2020), continuous learning approaches in pediatric patients (Hughes et al., 2021), clinical decision support tools for individualized dosing in pediatric patients (Frymoyer et al., 2020), application in Japanese patients (Matsumoto et al., 2022), and the importance of Bayesian approaches for faster and reliable monitoring (Aljutayli et al., 2022). While there are works related to MIPD in vancomycin treatment for Asian patients other than Japanese populations (Purwonugroho et al., 2012; Deng et al., 2013; Vu et al., 2019; Munir et al., 2021; Belavagi et al., 2022; Wei et al., 2022), in the Korean population, a study has been conducted in neonatal patients only (Lee et al., 2021).

In addition to dose optimization to avoid toxicity and resistance, an important aspect of vancomycin treatment is knowing when to stop dosing to avoid unnecessarily prolonged treatment (Rhee et al., 2020). This can be done by developing a pharmacodynamic (PD) model to predict the time course of anti-bacterial effects. While bacterial eradiation would be the most meaningful PD endpoint for that purpose, in routine clinical care settings where such information is usually not available, an alternative endpoint could be C-reactive protein (CRP). CRP plasma concentration, which is maintained between 1 and 10 mg/L, increases up to 100-fold within 2 h of the onset of inflammation and peaks almost within 48 h (Kindmark, 1972; Lelubre et al., 2013).

**FIGURE 1:**
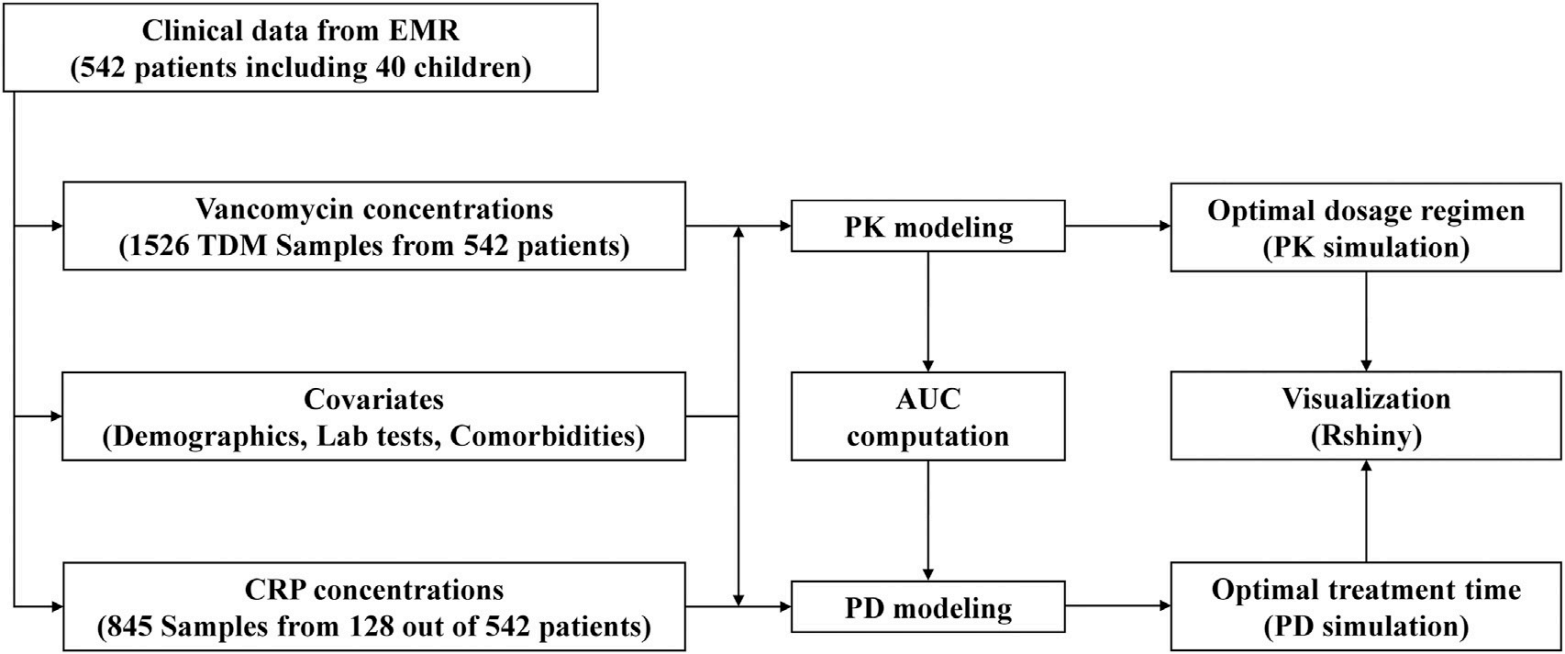
Study flow chart.

With this background, this study aims to i) develop a vancomycin population PK model and determine the optimal individualized dosage regimen within the MIPD framework, ii) develop a vancomycin PD model to identify the optimal treatment time, and iii) develop a web-based tool to visualize vancomycin PK and PD profiles for a selected dosage regimen applicable in clinical practice. Figure 1 illustrates the flow chart of this study.

## 2 Methods

### 2.1 Data collection

This was a retrospective study for PK and PD properties of vancomycin in 542 patients including 40 pediatric patients (<20 years), who received vancomycin intravenous IV) infusion at Severance Hospital in Seoul, Republic of Korea. The data were extracted from electronic medical records. The study encompassed patients who were treated with vancomycin and underwent Therapeutic Drug Monitoring (TDM) services. Patients lacking dosing history and concentration sampling time information were excluded from the analysis. Demographic information, laboratory test results such as creatinine and blood urea nitrogen (BUN), and comorbidity details were collected as potential covariates, as well as drug concentrations with 3 measurements per patient on average for PK analysis and CRP concentrations for PD analysis. PK and PD analyses were performed using NONMEM software version 7.5 (ICON Development Solutions, Dublin, Ireland) and exploratory data analysis was conducted using R software version 4.2.2.

### 2.2 Bioanalysis

Serum vancomycin levels were assessed using the kinetic interaction of microparticles in solution (KIMS) method on the Roche Cobas c702 analyzer (Roche, Basel, Switzerland). A competitive reaction takes place between the vancomycin-macromolecule conjugate and vancomycin in the serum to bind with the vancomycin antibody on the microparticles. The turbidity induced by the binding of the vancomycin conjugate to the antibody on the microparticles is measured photometrically, and this measurement is inhibited by the presence of vancomycin in the sample. The resulting turbidity is indirectly proportional to the amount of vancomycin present in the sample. The lower limit of quantitation for vancomycin is 4.0 μg/mL, determined as the lowest concentration that meets a total error goal of 20%.

CRP concentrations were determined through a particle-enhanced immunoturbidimetric assay on the Roche Cobas c702 as well. Serum creatinine levels were measured by a rate-blanked compensated kinetic Jaffe method on the Atellica CH 930 Analyzer (Siemens Healthcare Diagnostics, Marburg, Germany).

### 2.3 Basic PK model building

One- and two-compartment disposition models were tested for basic structural model since sparse samples near peak and trough concentrations were taken, where trough and peak concentrations were concentrations at the start and the end of IV infusion within one dosing interval, respectively. Systemic and inter-compartmental clearance and central and peripheral volume of distribution were parameterized using allometry scaling (N. H. Holford, 1996) as the following equations

$$TVV=\theta_{V}\times \left(\text{WT}/70\right)\times {\text{COV}}_{V}$$

$$TVCL=\theta_{\text{CL}}\times {\left(\text{WT}/70\right)}^{0.75}\times F_{\text{ren}}\times F_{\text{mat}}\times {\text{COV}}_{\text{CL}}$$

where WT is body weight, TVV and TVCL denote population typical value of volume of distribution (V) and clearance (CL), and $\theta_{V}$ and $\theta_{\text{CL}}$ denote population median values of V and CL for the subject with WT = 70 kg. The same allometry scaling was also applied to the inter-compartmental clearance (Q) and peripheral volume of distribution (Vp). The exponential model was applied to inter-individual variation of these parameters, and for the residual error, additive, proportional and combined models were compared.

### 2.4 Covariate model building

Assuming vancomycin clearance is affected by age-related renal function factor ( $F_{\text{ren}}$ ) and clearance maturation factor ( $F_{\text{mat}}$ ) associated with postmenstrual age (PMA) of up to 48 weeks (Anderson et al., 2007), covariate model building was conducted using the following model structure as a basis:

$$TVV=\theta_{V}\times \left(\text{WT}/70\right)\times {\text{COV}}_{V}$$

$$TVCL=\theta_{\text{CL}}\times {\left(\text{WT}/70\right)}^{0.75}\times F_{\text{ren}}\times F_{\text{mat}}\times {\text{COV}}_{\text{CL}}$$

where ${\text{COV}}_{V}$ and ${\text{COV}}_{\text{CL}}$ denote functions of additional covariates to be searched for V and CL, respectively. Then, $F_{\text{ren}}$ was formulated as (Anderson et al., 2007):

$$F_{\text{ren}}={\left(\text{CLCr}/{\text{CLCr}}_{\text{TV}}\right)}^{\lambda }$$

$$CLCr=R_{\text{Cr}}/\text{Cr}\times e^{\left(-k_{\text{tox}}∙t\right)}$$

$$R_{\text{Cr}}=64.2\times e^{\left(k_{\text{Cr}}∙\left(age-30\right)\right)}$$

where for the $F_{\text{ren}}$ model, CLCr is creatinine clearance, ${\text{CLCr}}_{\text{TV}}$ is the typical value of CLCr corresponding to the subject with age of 30 years old, and *λ* is an exponent parameter, for the CLCr model, Cr is plasma creatinine concentration, $R_{\text{Cr}}$ is Cr production rate and $k_{\text{tox}}$ means a rate constant to describe the reduced creatinine clearance due to vancomycin-induced nephrotoxicity which could reduce clearance, and for the $R_{\text{Cr}}$ model, 64.2 (mg⁄h) is the value of $R_{\text{Cr}}$ for the subject with age of 30 years old, and $k_{\text{Cr}}$ is a rate constant related to age (Cockcroft and Gault, 1976) such that $k_{\text{Cr}}$ > 0 for age <30 and $k_{\text{Cr}}$ ≤ 0 for age ≥30.

On the other hand, $F_{\text{mat}}$ , which applied to patients under 4 years old, was formulated as (N. Holford et al., 2013):

$$F_{\text{mat}}={\text{PMA}}^{\gamma }/\left({PMA50}^{\gamma }+{\text{PMA}}^{\gamma }\right)$$

where PMA50 denotes PMA where maturation reaches 50% of the adult clearance and *γ* is a steepness factor of sigmoid function.

After integrating these into clearance, potential covariates such as age, gender, BUN, and the history of hypertension, diabetes, renal diseases, cardiovascular disease, hematological diseases, pleural effusion and edema, and sepsis were tested based on pharmacological and physiological plausibility, as denoted by ${\text{COV}}_{V}$ and ${\text{COV}}_{\text{CL}}$ . Stepwise covariate model building was conducted with a likelihood ratio test based on the criteria of *p* < 0.01 (ΔOFV = 6.63, df = 1) for forward selection and *p* < 0.001 (ΔOFV = 10.82, df = 1) for backward deletion, where OFV means objective function value of NONMEM.

### 2.5 PD model building

To characterize the dynamics of CRP for the time delay between infection and biomarker level change in the plasma, a semi-mechanistic model with proliferation and transit compartments was attempted which was formulated as below:

$$\frac{\text{dProl}}{\text{dt}}=K_{\text{in}}\times \left(1+S_{\text{CRP}}∙D\right)-K_{\text{out}}\times Prol\;;\;Proliferation$$

$$\frac{dTran1}{\text{dt}}=k_{\text{tr}}\times \left(Prol-Tran1\right)\;;\;Transit\;1$$

$$\frac{dTran2}{\text{dt}}=k_{\text{tr}}\times \left(Tran1-Tran2\right)\;;\;Transit\;2$$

$$\frac{\text{dCRP}}{\text{dt}}=k_{\text{tr}}\times Tran2-k_{\text{CRP}}\times CRP\;;\;Circulation$$

Prol is the proliferation compartment, with K_in_ and K_out_ denoting production and degradation rate constant, respectively, which was assumed to be K_in_ = K_out_ = $k_{\text{tr}}$ for numerical simplicity, Tran1 and Tran2 are the transition compartments, with $k_{\text{tr}}$ denoting a transit rate constant, and CRP is the circulating compartment, with $k_{\text{CRP}}$ denoting an elimination rate constant. $S_{\text{CRP}}$ is a scaling factor and its effect is proportional to disease severity (D), which was estimated as the following equations where α is a scaling factor.

$$\frac{\text{dD}}{\text{dt}}=k_{D}\times D\times \left(1-E_{\text{Drug}}\right)$$

$$E_{\text{Drug}}=\alpha \times AUC$$

AUC obtained from the developed PK model, $E_{\text{Drug}}$ means drug effect and $k_{D}$ is a first-order progression rate constant. To find covariates for PD model parameters, a stepwise selection was done with a likelihood ratio test based on the criteria of *p* < 0.05 (ΔOFV = 3.84, df = 1) for forward selection and *p* < 0.01 for backward deletion.

### 2.6 Model evaluation

To select the final model, the tested models were compared based on OFV or AIC values and the precision of parameter estimates. The selected model was then evaluated with goodness-of-fit plots such as observation *versus* model prediction (PRED), and conditional weighted residual (CWRES) *versus* PRED. Subsequently, a visual predicted check (VPC) was conducted for the validation of the selected model, ensuring that collected drug concentrations fall within the 95% confidence interval of the 2.5^th^ percentile, median, and 97.5^th^ percentile of predicted drug concentrations.

### 2.7 Simulation for optimal dosage regimen

Using the final PK model, simulations were conducted to explore optimal dosage regimens for sub-populations stratified by selected covariates. To achieve this, for the m selected covariates, the range from the minimum to the maximum values for each covariate i was divided into equi-spaced intervals $N_{i}$ , resulting in a total of $N_{1}\times N_{2}\times ∙∙∙\times N_{m}$ scenarios of covariate pairs.

These scenarios were simulated using typical values for intravenous administration four times a day (QID). The objective was to determine the optimal dosage regimen that meets the criteria of achieving a trough concentration closest to the target value, which was set at 7 mg/L for children under the age of 4, 10 mg/L for those aged 4 to 19, and 15 mg/L for adults. Additionally, the peak concentrations were required to remain below the toxic level of 40 mg/L across all age groups. These criteria were based on findings from previous studies (Rybak M. et al., 2009; Rybak et al., 2020).

### 2.8 Simulation for the time course of anti-bacterial effects

Using the developed PD model and the selected covariates, the time course of CRP plasma concentrations was simulated by varying the dose, infusion rate, and inter-dose interval in order to predict the optimal time to discontinue treatment and prevent unnecessary overtreatment.

### 2.9 Development of a web-based tool

A web-based application was developed using R Shiny to visualize the simulated time course of plasma concentrations of vancomycin and CRP, which is based on the developed PK-PD model and the selected dosage regimen. This application allows users to visualize the predicted individual PK-PD profile for a specific dosage regimen.

## 3 Results

### 3.1 Patient information

A total of 542 hospitalized patients were included in the PK model building, with 22 aged under 4, 18 aged 4 to 19, and 502 adults. Among them, 128 patients were eligible for the PD analysis. Vancomycin was administered at doses ranging from 500 to 1,500 mg, with dose intervals ranging from 6 to 24 h. The patient population spanned from neonates to the elderly, and 40 pediatric patients were included in both PK and PD analyses. The average length of hospital stay was 20 days, with a minimum of 2 days and a maximum of 113 days. The hospitalization of up to 113 days was due to a secondary infection caused by pneumonia, which necessitated the extension of vancomycin treatment. Detailed demographic information for the patients can be found in Tables 1, 2.

**TABLE 1:** Demographic of patients who were included in the PK analysis.

The total number of patients = 542	Samples (vancomycin concentrations) = 1,526	
Continuous Variable	Median (Min, Max)	
Age (year)	60 (0, 93)	
PMA (month)	70 (39, 232)	
Weight (kg)	59 (2.6, 106)	
Albumin (g/dL)	2.9 (1, 4.4)	
Total protein (g/dL)	5.7 (3.4, 8.4)	
Creatinine (mg/dL)	0.7 (0.2, 12.9)	
BUN (mg/dL)	15.25 (1.5, 141.5)	
Vancomycin concentration (mg/L)	23.8 (1.7, 99)	

Categorical Variable	(Number, %)	(Number, %)
Sex	Male (319, 58.9)	Female (223, 41.1)
Hypertension	No (258, 47.6)	Yes (284, 52.4)
Diabetes	No (388, 71.6)	Yes (154, 28.4)
Neutropenia	No (523, 89.9)	Yes (19, 3.5)
Sepsis	No (447, 82.5)	Yes (95, 17.5)
Hematological malignancy	No (420, 77.5)	Yes (122, 22.5)
Pleural effusion and edema	No (493, 91)	Yes (49, 9.0)
Cardiovascular disease	No (286, 52.8)	Yes (256, 47.2)
Renal diseases	No	(395, 72.9)
	Acute kidney disease	(49, 9.04)
	Chronic kidney disease	(48, 8.86)
	Others	(50, 9.23)

PMA, postmenstrual age; BUN, blood urea nitrogen; SD, standard deviation; Min, Minimum; Max, Maximum.

**TABLE 2:** Demographic of patients who were included in the PD analysis.

The number of patients = 128	Samples (CRP measurements) = 845	
Continuous Variable	Median (Min, Max)	
Age (year)	63 (9, 87)	
Weight (kg)	57.15 (31.5, 106)	
Albumin (g/dL)	2.8 (1.7, 4.0)	
Total protein (g/dL)	5.55 (3.9, 8.4)	
Creatinine (mg/dL)	0.79 (0.2, 10.35)	
BUN (mg/dL)	18.05 (1.9, 141.5)	
CRP concentration (mg/L)	73 (0.4, 479.2)	

Categorical Variable	(Number, %)	(Number, %)
Sex	Male (79, 61.7)	Female (49, 38.3)
Pneumonia	No (87, 68.0)	Yes (41, 32.0)
Hypertension	No (46, 35.9)	Yes (82, 64.1)
Diabetes	No (76, 59.4)	Yes (52, 40.6)
Neutropenia	No (126, 98.4)	Yes (2, 1.6)
Sepsis	No (101, 78.9)	Yes (27, 21.1)
Hematological malignancy	No (115, 89.8)	Yes (13, 10.2)
Pleural effusion and edema	No (117, 91.4)	Yes (11, 8.6)
Cardiovascular disease	No (63, 49.2)	Yes (65, 50.8)
Renal diseases	No	(87, 68)
	Acute kidney disease	(13, 10.9)
	Chronic kidney disease	(14, 10.2)
	Others	(14, 10.9)
Co-infected bacterial species	None	(109, 85.2)
	Other *Staphylococcus* species	(18, 14.1)
	*Streptococcus* species	(1, 0.8)

CRP, C-reactive protein, PMA, postmenstrual age; BUN, blood urea nitrogen; SD, standard deviation; Min, Minimum; Max, Maximum.

### 3.2 PK model

Various models were tested using the collected vancomycin concentration data, and a 2-compartment model incorporating allometry scaling was selected as the basic model based on the OFV value. For covariates, gender, BUN, and the history of diabetes and renal disease were selected for ${\text{COV}}_{\text{CL}}$ and age for ${\text{COV}}_{V}$ as follows:

$${\text{COV}}_{\text{CL}}=e^{\left(k_{\text{BUN}}∙\left(BUN-15\right)\right)}\times \left(1+\theta_{\text{FEM}}∙FEM\right)\times \left(1+\theta_{\text{DM}}∙DM\right)\times \left(1+\theta_{\text{REN}}∙REN\right)$$

$${\text{COV}}_{V}=e^{\left(k_{V}∙\left(age-40\right)\right)}$$

where FEM = 1 for female and 0 for male, DM = 1 for diabetes and 0 for no diabetes, and REN = 1 for renal disease and 0 for no renal disease.

**FIGURE 2:**
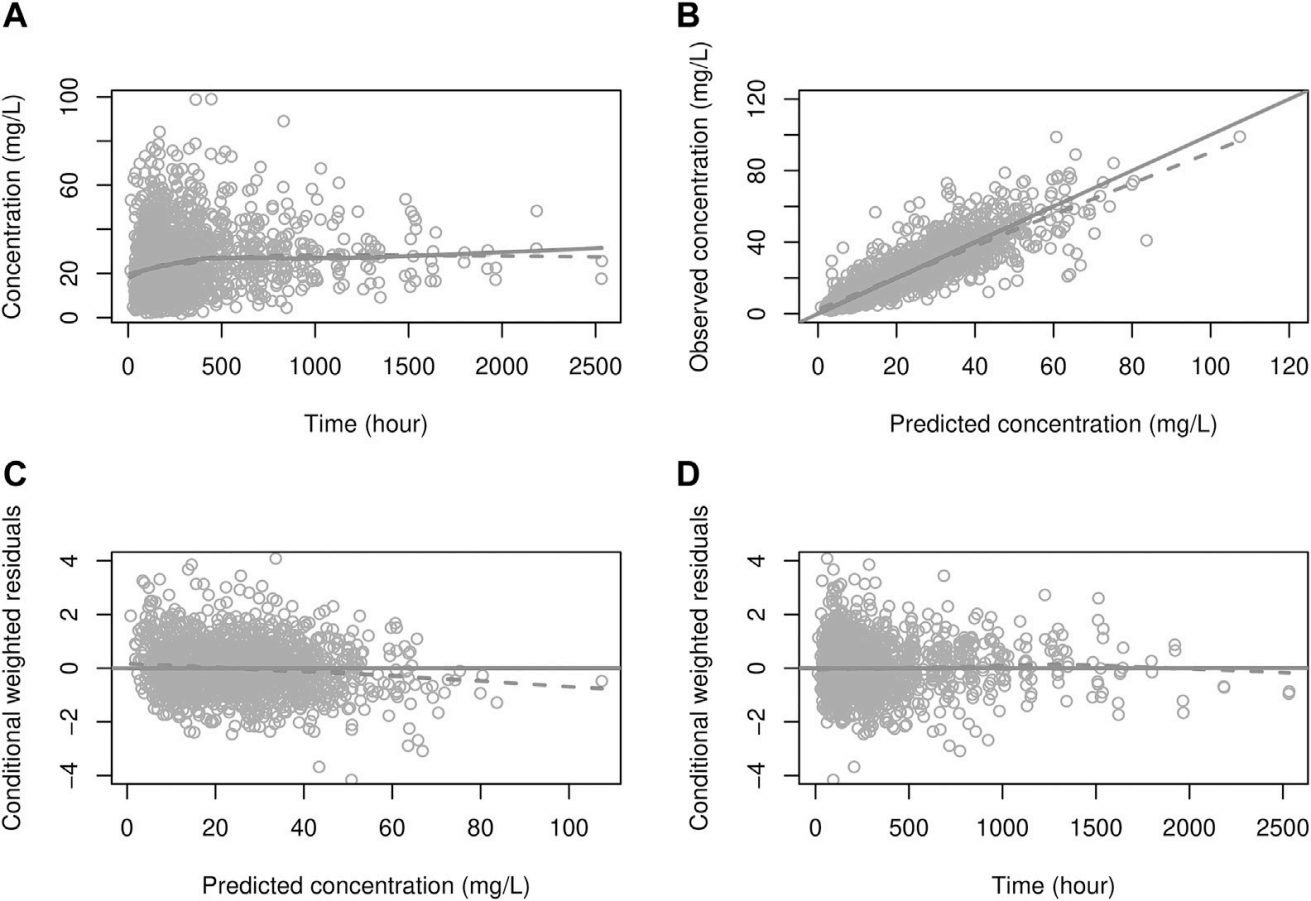
Goodness-of-fit plot for the PK model **(A)** solid line: prediction; dots: observations, dashed line: smooth (of observations), **(B)** solid line: identity line; dashed line: smooth, **(C, D)** solid line: zero residual line; dashed line: smooth.

**FIGURE 3:**
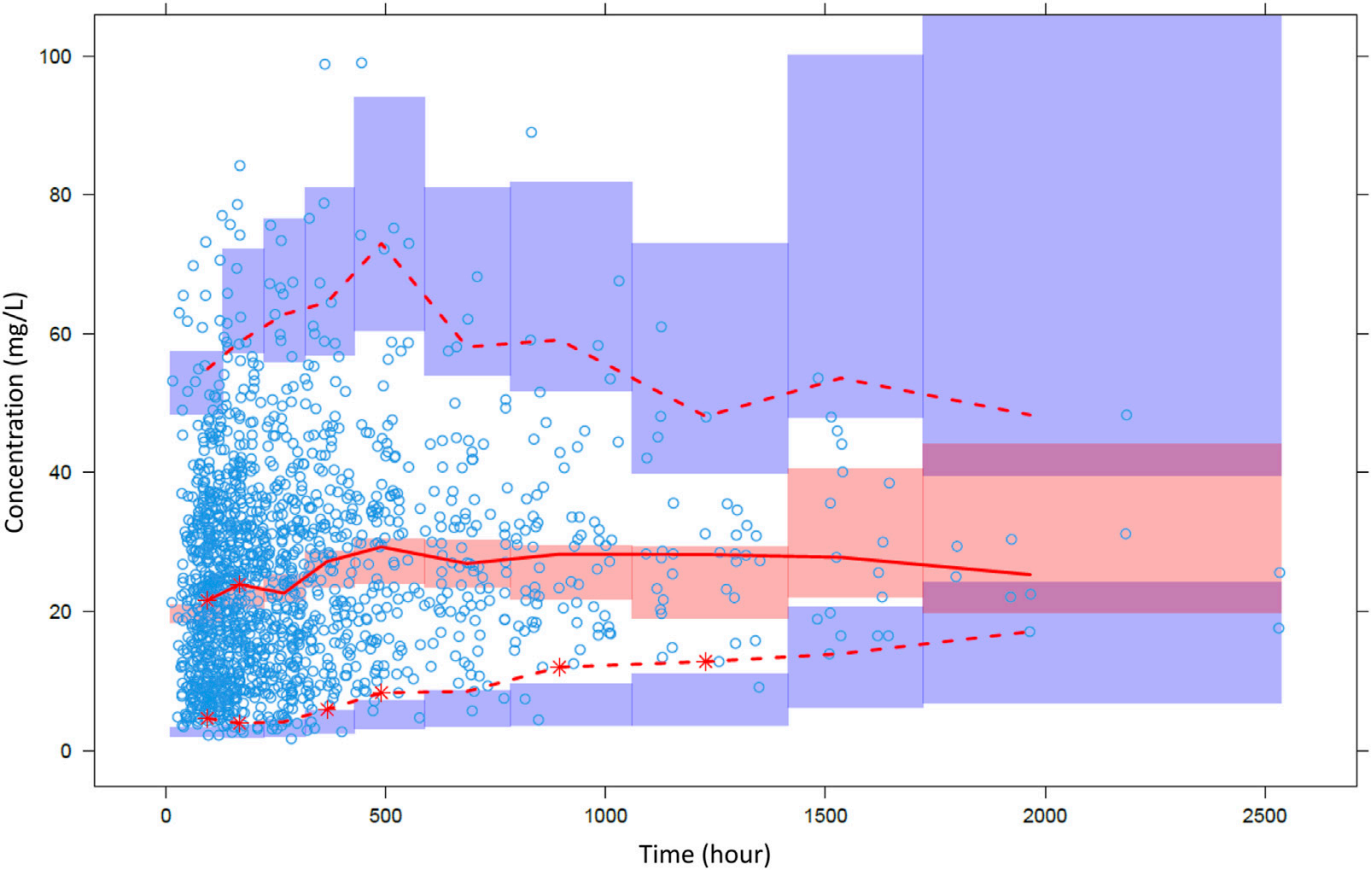
Visual predictive check of the final pharmacokinetic model. Open circles are observations and lines are 2.5^th^, median and 97.5^th^ percentiles of observations. Colored area means confidence interval of each prediction percentile.

The incorporation of vancomycin-induced nephrotoxicity $\left(k_{\text{tox}}\right.$ ) into the model significantly improved model prediction (*p* < 0.0001). Vancomycin CL exhibited a gradual increase with BUN levels up to 15 mg/dL, followed by a subsequent decrease. In the final model, median values of CL and V were estimated to be 4.31 L/h and 38.6 L, respectively. The details of final parameter estimates are presented in Table 3, and these values were consistent with findings from other studies. Regarding the relative standard error (RSE), except for *γ*, most parameters, demonstrated reliable estimation. Goodness-of-fit plots of the PK model are shown in Figure 2, revealing the absence of noticeable trends. VPC plots in Figure 3 indicated that the majority of observations fell within the 95% confidence intervals of the predictions. In these figures, the vancomycin concentration data depicted represent observations during repeated dosing after hospitalization. The two concentration measurements at 2,500 h correspond to peak and trough samples obtained from the patient who was hospitalized for 113 days or 2,712 h.

**TABLE 3:** Parameter estimates of the final pharmacokinetic model.

Parameter	Population estimate (%RSE)
Structural parameter	
*θ*_CL_ (L/h)	4.32 (4.33)
*θ*_V_ (L)	38.6 (2.69)
*θ*_Q_ (L/h)	3.93 (9.31)
*θ*_V2_ (L)	66.8 (9.78)
(Creatinine clearance related parameter)	
$k_{\text{Cr}}$ (if age ≥30) (yr^-1^)	−0.0127 (14.3)
$k_{\text{Cr}}$ (if age <30) (yr^-1^)	0.0193 (37.9)
λ	0.655 (3.66)
(Maturation related parameter)	
PMA50	43.9 (16.3)
γ	2.08 (63.5)
(Nephrotoxicity related parameter)	
$k_{\text{tox}}$ (day^-1^)	0.00598 (19.9)
(Covariate related parameter)	
$k_{V}$	0.00957 (8.88)
$k_{\text{BUN}}$	−0.00874 (13.2)
$\theta_{\text{REN}}$	−0.237 (13.0)
$\theta_{\text{FEM}}$	−0.199 (12.7)
$\theta_{\text{DM}}$	−0.151 (22.3)
Between subject variability	
ω^2^_CL_ (CV(%))	29.1 (4.18)
ω^2^_V2_ (CV(%))	101 (8.04)
Residual variability	
σ^2^_proportional trough_ (CV %))	17.8 (5.57)
σ^2^_additive trough_ (mg/L)	0.956 (23.0)
σ^2^_proportional peak_ (CV %))	11.0 (11.6)
σ^2^_additive peak_ (mg/L)	4.47 (8.59)

CL, clearance; V, volume of distribution; Q, Inter-compartmental clearance; V2, peripheral volume of distribution; $k_{\text{Cr}}$ , Age related rate constant of renal function; λ , Exponent of renal function; PMA50, Postmenstrual age at 50% organ maturation; γ, Steepness factor of sigmoid function of maturation; $k_{\text{tox}}$ , Nephrotoxicity related rate constant; $k_{V}$ , Covariate coefficient of aging effect; $k_{\text{BUN}}$ , Covariate coefficient of blood urea nitrogen effect; $\theta_{\text{REN}}$ , Covariate coefficient of renal disease effect; $\theta_{\text{FEM}}$ , Covariate coefficient of sex effect; $\theta_{\text{DM}}$ , Covariate coefficient of diabetes effect; RSE, relative standard error; CV, coefficient of variance.

### 3.3 PD model

A semi-mechanistic model with one proliferation compartment and two transit compartments was selected as the structural model. Among the covariates, the presence of pneumonia had a significant effect on the transit rate constant as in the following equation, where PNE = 1 indicates pneumonia and 0 indicates no pneumonia.

$$k_{\text{tr}}=\theta_{k_{\text{tr}}}+\theta_{\text{PNE}}∙PNE$$

**FIGURE 4:**
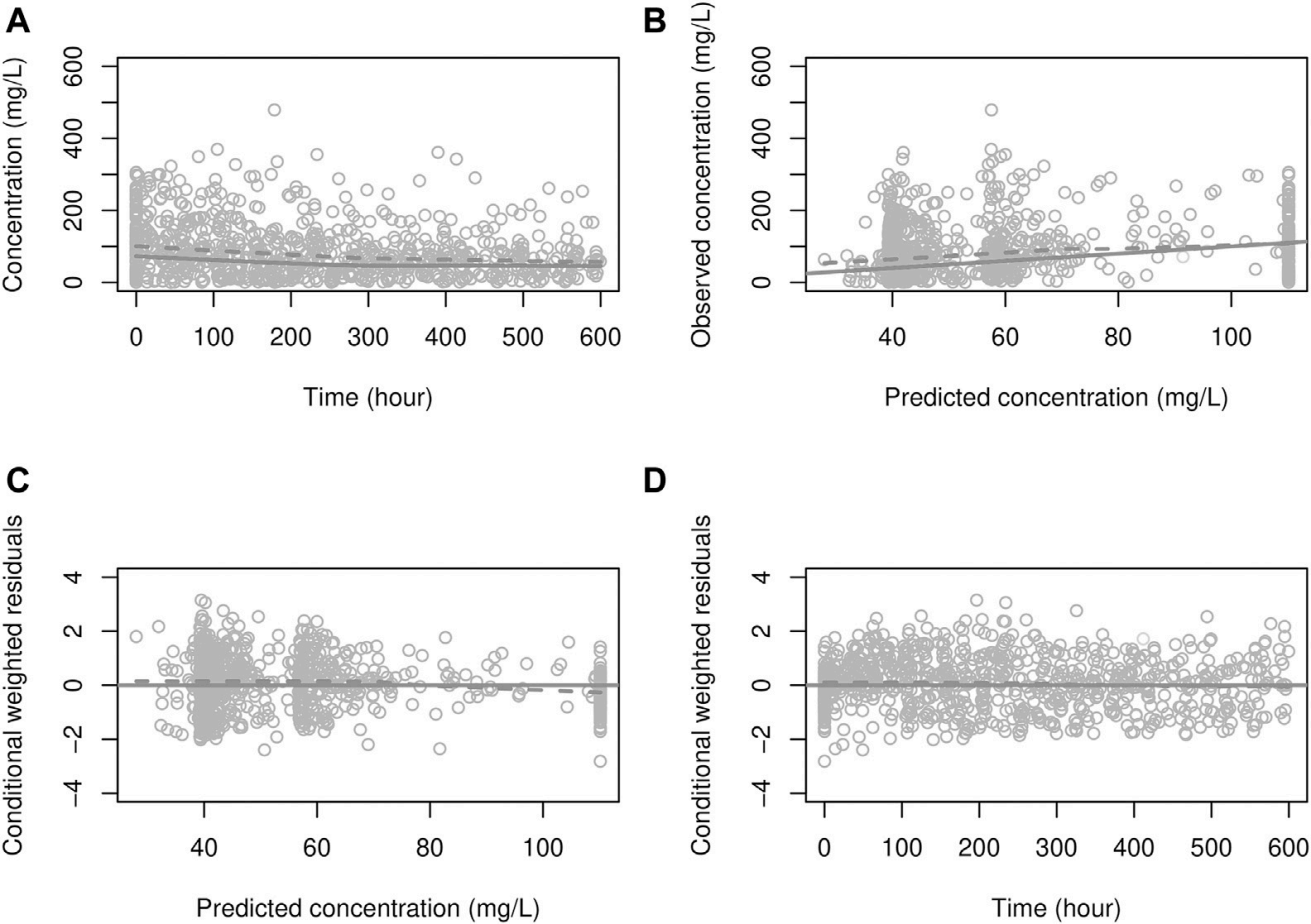
Goodness-of-fit plot for the PD model. **(A)** solid line: prediction; dots: observations, dashed line: smooth (of observations), **(B)** solid line: identity line; dashed line: smooth, **(C, D)** solid line: zero residual line; dashed line: smooth.

The final parameter estimates are presented in Table 4. Except for $k_{D}$ and CRP_0_, the initial value of CRP, between-subject variability (BSV) could not be obtained due to numerical difficulties. Consequently, $k_{\text{CRP}}$ was fixed at 0.0365 h^−1^ based on the prior knowledge that CRP’s half-life was 19 h (Vigushin et al., 1993). Mean transit times for pneumonia and non-pneumonia patients were 6.65 and 9.62 days, respectively. Figure 4 presented the goodness-of-fit plots where no obvious trends were observed, indicating, overall, the model adequately describes CRP concentrations.

**TABLE 4:** Parameter estimates of the final pharmacodynamic model.

Parameter	Population estimate (%RSE)
Structural parameter	
$\theta_{k_{\text{tr}}}$ (h^-1^)	0.0129 (4.90)
$\theta_{\text{PNE}}$ (h^-1^)	0.0058 (17.2)
$k_{\text{CRP}}$ (h^-1^)	0.365 FIX
CRP_0_ (mg/L)	110 (8.4)
$k_{D}$ (h^-1^)	0.00192 (29.2)
α	0.000239 (8.12)
S_CRP_	102 (6.56)
Between subject variability	
ω^2^_KD_ (CV(%))	147.6 (19.5)
ω^2^_CRP0_ (CV(%))	107.2 (7.65)
Residual variability	
σ^2^_proportional_ (CV %))	54.9 (2.71)

*k*_*tr*_, Transit rate constant; $\theta_{PNE}$ , Covariate coefficient of pneumonia effect; $k_{CRP}$ , elimination rate constant; *CRP*_0_, Initial value of C-reactive protein, $k_{D}$ , Progression rate constant; α, Scaling factor for drug effect; S_CRP_, scaling factor for proliferation; RSE, relative standard error; CV, coefficient of variance.

### 3.4 Simulation for optimal dosage regimen

Considering the range of covariates observed in the collected data, a total of 384 scenarios were simulated for adult patients, using 4 Cr levels, 4 BUN levels, 4 age levels, and 6 body weight (WT) levels. For pediatric patients, a total of 656 scenarios were simulated. Among these, 336 scenarios were designed for patients aged 4 years or younger, incorporating 7 PMA levels, 3 WT levels, 4 Cr levels, and 4 BUN levels. An additional 320 scenarios were created for patients aged over 4 years, employing 4 age levels, 5 WT levels, 4 Cr levels, and 4 BUN levels.

**FIGURE 5:**
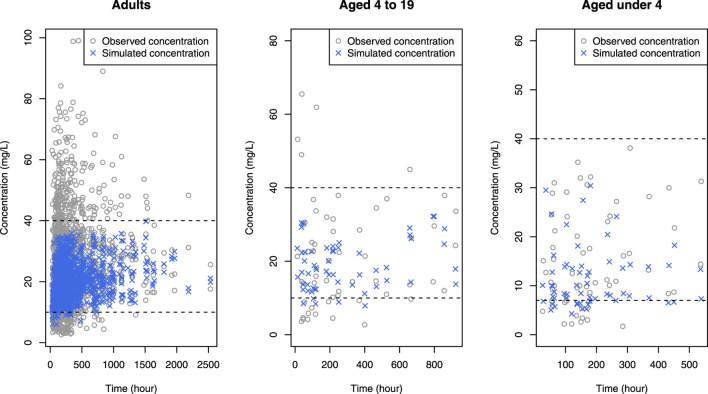
Simulated concentrations (blue crosses) obtained when the optimal dose was given to each patient in the data, superimposed by observed concentrations (gray open circles), where black horizontal dashed lines mean upper and lower limit of therapeutic range.

Daily doses were explored in increments of 0.2 g (0.05 g per dose; QID) for adults, and in increments of 5 mg/kg (1.25 mg/kg per dose; QID) for pediatrics. Detailed results are presented in Table 5 for adults, Table 6 for pediatric patients and Table 7 for those aged under 4. The MIPDs were provided for each combination of covariate values, and the shading increased with the dose. For precision dosing presented in the tables, the covariate model for ${\text{COV}}_{\text{CL}}$ indicated that dose reductions of 23%, 15%, and 20% were necessary for patients with renal diseases, diabetes and females, respectively. In simulation results, the optimal dose increased with weight, while it decreased with Cr and BUN levels. Regarding age, the dose exhibited an upward trend with age until 40, followed by a subsequent decrease. Regarding the attainment of target concentrations, it was confirmed that simulated concentrations given optimal doses were within the therapeutic range across all age groups. Figure 5 illustrates that when concentrations of the study patients were simulated using the optimal dose from a virtual patient with the most analogous covariates to the study patient, the majority of simulated concentrations lied within the therapeutic range. In contrast, a significant portion of observed concentrations fell outside this range.

**TABLE 5:** Simulation results for optimal daily dose in adults (g/day).

Age (year)	Weight (kg)	Creatinine (mg/dL)																			
		0.5				1.0				1.5				2.0				>2.5			
		BUN (mg/dL)																			
		10	20	30	>40	10	20	30	>40	10	20	30	>40	10	20	30	>40	10	20	30	>40
20	40	2.2	2	1.8	1.6	1.2	1.2	1	1	1	0.8	0.8	0.6	0.8	0.6	0.6	0.6	0.6	0.6	0.6	0.6
	50	2.6	2.4	2	1.8	1.4	1.4	1.2	1	1	1	1	0.8	0.8	0.8	0.8	0.6	0.8	0.8	0.6	0.6
	60	3	2.6	2.4	2.2	1.6	1.6	1.4	1.2	1.2	1.2	1	1	1	1	0.8	0.8	0.8	0.8	0.8	0.8
	70	3.2	3	2.6	2.4	1.8	1.6	1.6	1.4	1.4	1.2	1.2	1	1.2	1	1	1	1	1	0.8	0.8
	80	3.6	3.2	2.8	2.6	2	1.8	1.6	1.6	1.6	1.4	1.2	1.2	1.2	1.2	1	1	1.2	1	1	0.8
	90	4	3.4	3.2	2.8	2.2	2	1.8	1.6	1.6	1.6	1.4	1.2	1.4	1.2	1.2	1	1.2	1.2	1	1
40	40	2.2	2	1.8	1.6	1.2	1.2	1	1	1	0.8	0.8	0.8	0.8	0.8	0.6	0.6	0.6	0.6	0.6	0.6
	50	2.6	2.4	2	1.8	1.4	1.4	1.2	1.2	1.2	1	1	0.8	1	0.8	0.8	0.8	0.8	0.8	0.6	0.6
	60	3	2.6	2.4	2.2	1.6	1.6	1.4	1.2	1.2	1.2	1	1	1	1	0.8	0.8	1	0.8	0.8	0.8
	70	3.2	3	2.6	2.4	1.8	1.8	1.6	1.4	1.4	1.4	1.2	1	1.2	1	1	1	1	1	0.8	0.8
	80	3.6	3.2	2.8	2.6	2	1.8	1.8	1.6	1.6	1.4	1.4	1.2	1.4	1.2	1.2	1	1.2	1	1	1
	90	3.8	3.4	3.2	2.8	2.2	2	1.8	1.8	1.8	1.6	1.4	1.4	1.4	1.4	1.2	1.2	1.2	1.2	1	1
60	40	1.8	1.6	1.4	1.2	1	1	0.8	0.8	0.8	0.8	0.6	0.6	0.6	0.6	0.6	0.6	0.6	0.6	0.4	0.4
	50	2	1.8	1.6	1.4	1.2	1	1	1	1	0.8	0.8	0.8	0.8	0.8	0.6	0.6	0.6	0.6	0.6	0.6
	60	2.2	2	1.8	1.6	1.4	1.2	1.2	1	1	1	1	0.8	0.8	0.8	0.8	0.8	0.8	0.8	0.6	0.6
	70	2.6	2.2	2	1.8	1.6	1.4	1.2	1.2	1.2	1	1	1	1	1	0.8	0.8	0.8	0.8	0.8	0.8
	80	2.8	2.6	2.2	2	1.8	1.6	1.4	1.4	1.4	1.2	1.2	1	1.2	1	1	1	1	1	0.8	0.8
	90	3	2.8	2.4	2.2	1.8	1.8	1.6	1.4	1.4	1.4	1.2	1.2	1.2	1.2	1	1	1	1	1	1
80	40	1.4	1.2	1.2	1	0.8	0.8	0.8	0.6	0.6	0.6	0.6	0.6	0.6	0.6	0.4	0.4	0.6	0.4	0.4	0.4
	50	1.6	1.4	1.4	1.2	1	1	0.8	0.8	0.8	0.8	0.6	0.6	0.6	0.6	0.6	0.6	0.6	0.6	0.6	0.6
	60	1.8	1.6	1.6	1.4	1.2	1	1	1	1	0.8	0.8	0.8	0.8	0.8	0.6	0.6	0.8	0.6	0.6	0.6
	70	2	1.8	1.6	1.6	1.2	1.2	1.2	1	1	1	0.8	0.8	0.8	0.8	0.8	0.8	0.8	0.8	0.8	0.6
	80	2.2	2	1.8	1.8	1.4	1.4	1.2	1.2	1.2	1	1	1	1	1	0.8	0.8	0.8	0.8	0.8	0.8
	90	2.4	2.2	2	1.8	1.6	1.4	1.4	1.2	1.2	1.2	1.2	1	1	1	1	1	1	1	0.8	0.8

BUN, Blood urea nitrogen. *The presented optimal dose needs to be reduced by 15% for diabetes, 23% for renal diseases, and 20% for female.

**TABLE 6:** Simulation results for optimal weight-normalized daily dose in pediatrics (mg/kg/day).

Age (year)	Weight (kg)	Creatinine (mg/dL)															
		0.5				1.0				1.5				>2.0			
		BUN (mg/dL)															
		10	15	20	>25	10	15	20	>25	10	15	20	>25	10	15	20	>25
6	17	50	45	40	40	25	25	20	20	20	15	15	15	15	15	15	10
	19	45	45	40	40	25	25	20	20	15	15	15	15	15	15	10	10
	21	45	40	40	35	25	20	20	20	15	15	15	15	15	15	10	10
	23	45	40	40	35	25	20	20	20	15	15	15	15	15	10	10	10
	25	40	40	35	35	20	20	20	20	15	15	15	15	15	10	10	10
10	27	45	40	40	35	25	20	20	20	15	15	15	15	15	15	10	10
	31	40	40	35	35	20	20	20	20	15	15	15	15	15	10	10	10
	35	40	35	35	35	20	20	20	20	15	15	15	15	10	10	10	10
	39	40	35	35	30	20	20	20	15	15	15	15	15	10	10	10	10
	43	35	35	30	30	20	20	20	15	15	15	15	10	10	10	10	10
14	40	40	40	35	35	20	20	20	20	15	15	15	15	10	10	10	10
	46	40	35	35	30	20	20	20	15	15	15	15	15	10	10	10	10
	52	35	35	30	30	20	20	20	15	15	15	15	10	10	10	10	10
	58	35	35	30	30	20	20	15	15	15	15	15	10	10	10	10	10
	67	35	30	30	30	20	15	15	15	15	15	10	10	10	10	10	10
18	45	40	40	35	35	20	20	20	20	15	15	15	15	15	10	10	10
	55	40	35	35	30	20	20	20	15	15	15	15	15	10	10	10	10
	65	35	35	30	30	20	20	15	15	15	15	15	10	10	10	10	10
	75	35	30	30	30	20	20	15	15	15	15	10	10	10	10	10	10
	85	35	30	30	25	20	15	15	15	15	15	10	10	10	10	10	10

BUN, Blood urea nitrogen. *The presented optimal dose needs to be reduced by 15% for diabetes, 23% for renal diseases, and 20% for female.

**TABLE 7:** Simulation results for optimal weight-normalized daily dose in pediatrics aged under 4 (mg/kg/day).

PMA (week)	Weight (kg)	Creatinine (mg/dL)															
		0.3				0.6				0.9				>1.2			
		BUN (mg/dL)															
		5	10	15	>20	5	10	15	>20	5	10	15	>20	5	10	15	>20
40	3	30	25	25	25	15	15	15	15	10	10	10	10	10	10	10	10
	3.5	30	25	25	25	15	15	15	15	10	10	10	10	10	10	10	10
	4	25	25	25	20	15	15	15	10	10	10	10	10	10	10	10	5
66	7	45	40	40	35	20	20	20	20	15	15	15	15	10	10	10	10
	8	40	40	35	35	20	20	20	15	15	15	15	10	10	10	10	10
	9	40	35	35	30	20	20	20	15	15	15	15	10	10	10	10	10
92	9	55	50	45	40	25	25	20	20	20	15	15	15	15	15	10	10
	10.5	50	45	45	40	25	25	20	20	15	15	15	15	15	10	10	10
	12	45	45	40	40	25	20	20	20	15	15	15	15	15	10	10	10
118	10	60	55	50	45	30	25	25	25	20	20	15	15	15	15	15	10
	12	55	50	45	45	25	25	25	20	20	15	15	15	15	15	15	10
	14	50	50	45	40	25	25	20	20	15	15	15	15	15	15	10	10
144	11	60	55	50	50	30	25	25	25	20	20	15	15	15	15	15	15
	13	55	55	50	45	25	25	25	20	20	15	15	15	15	15	15	10
	15	55	50	45	45	25	25	25	20	20	15	15	15	15	15	10	10
196	12.5	65	60	55	50	30	30	25	25	20	20	20	15	15	15	15	15
	14.5	60	55	50	45	30	25	25	25	20	20	15	15	15	15	15	15
	16.5	55	50	50	45	25	25	25	20	20	15	15	15	15	15	15	10
248	14	65	60	55	50	30	30	25	25	20	20	20	15	15	15	15	15
	16.5	60	55	50	45	30	25	25	25	20	20	15	15	15	15	15	15
	19	55	50	50	45	25	25	25	20	20	15	15	15	15	15	15	10

BUN, blood urea nitrogen; PMA, postmenstrual age. *The presented optimal dose needs to be reduced by 23% for renal diseases and 20% for female.

### 3.5 Simulation for the time course of anti-bacterial effects

**FIGURE 6:**
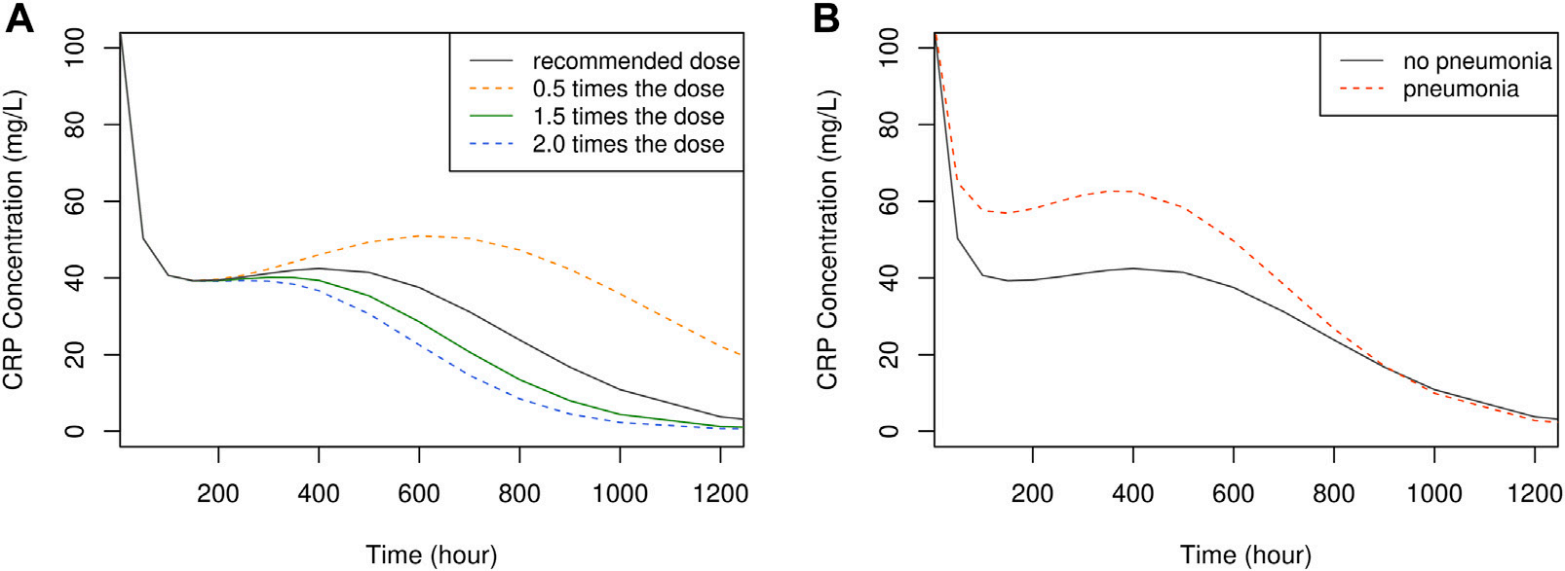
The time course of simulated CRP concentration, plotted by dose **(A)** and the presence of pneumonia **(B)**.

The influence of dose and pneumonia on CRP concentration-time profile is illustrated in Figure 6. No dose-dependent differences were observed up to approximately 240 h or 10 days. However, after this point, CRP levels exhibited a faster decline with higher doses, returning to the normal range of 1–10 mg/L at around 1,100 h or 46 days with the recommended dose. This suggests that discontinuing vancomycin treatment at 46 days is viable to prevent unnecessary overtreatment. It is worth noting that the extended time required for CRP levels to normalize can be attributed to the prolonged hospitalization period experienced by the study patients.

### 3.6 Development of a web-based tool

**FIGURE 7:**
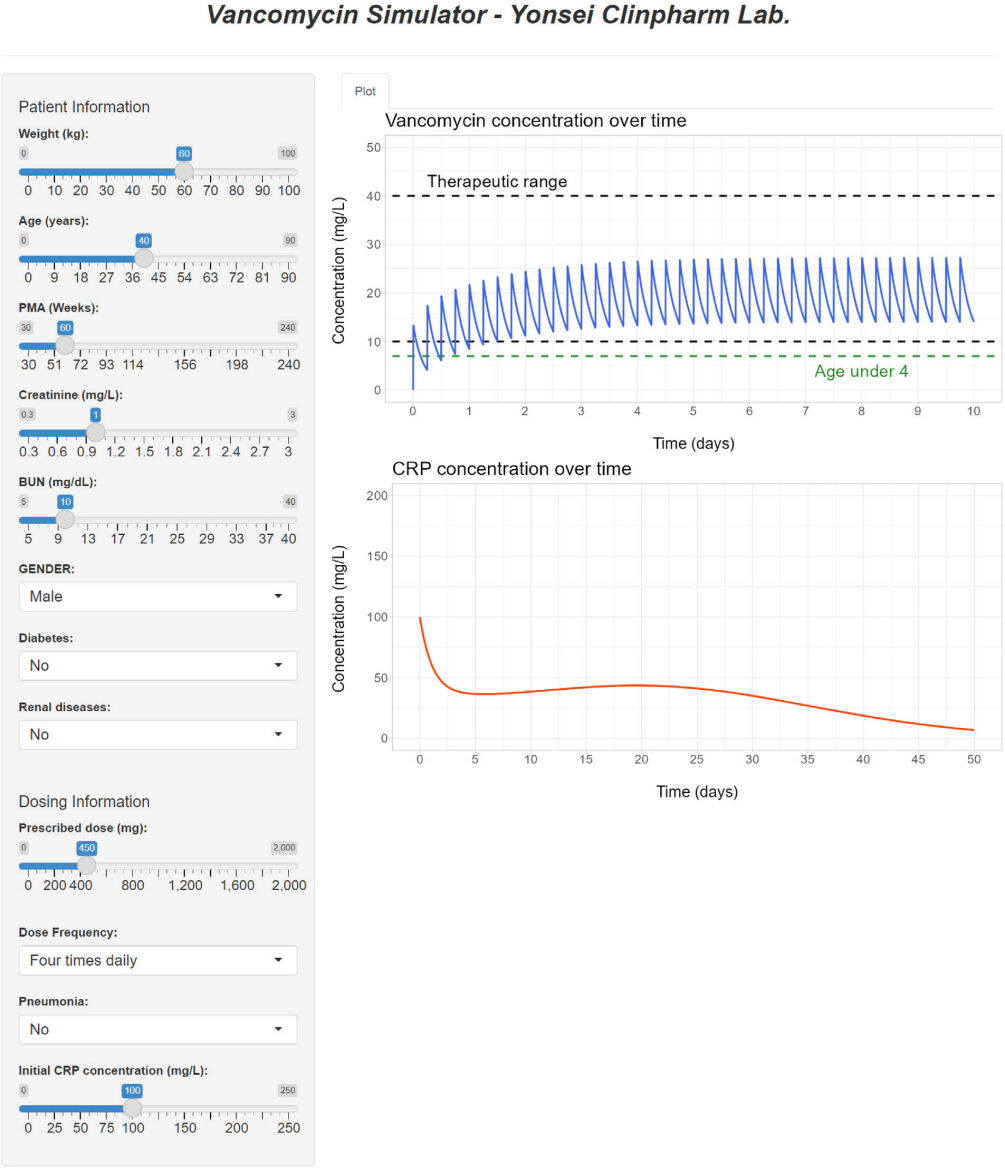
R Shiny application for vancomycin dose optimization and visualization of simulated PK and PD profiles.

The interface of the developed R Shiny tool for vancomycin dose optimization is showcased in Figure 7. This tool offers the capability to optimize the necessary dose for a patient to attain the desired drug concentration based on their individual covariates, achieved through the adjustment of the dose and/or dosing interval. By providing visualizations of drug concentration-time profiles, this tool serves as an alternative to the nomogram approach detailed in Tables 5–7. In addition to simulating drug concentrations, the application also facilitates the visualization of simulated CRP plasma concentrations that correspond to the employed dose, dosing rate, and covariates utilized in the PK simulation.

## 4 Discussion

The primary objective of this study was to present the optimal dosage regimen and treatment duration for Korean patients undergoing vancomycin treatment by utilizing a PK-PD model. Prior research within the framework of vancomycin dose adjustment using TDM has provided dosing guidelines based on peak and trough concentrations, considering factors such as AUC, MIC, and patient covariates (Rybak et al., 2020). However, these simplistic TDM-based approaches often fall short of offering truly personalized vancomycin therapy tailored to individual patient characteristics. In light of this limitation, PK model-based studies have delved into dose optimization (Anderson et al., 2007; Yamamoto et al., 2009; Deng et al., 2013; Abdel Hadi et al., 2016; Goti et al., 2018). However, it is a challenge to come across model-based endeavors that systematically develop optimal dosage regimens spanning all age groups and encompassing a diverse range of covariate effects.

Our PK model introduced $F_{\text{ren}}$ , which was formulated using CLCr, as an alternative to established methods such as MDRD or Schwartz’s formula or CKD-EPI equation. This decision was rooted in the limitations and imprecision of MDRD for pediatric patients below 12 years (Pierrat et al., 2003; Zachwieja et al., 2015) and the inapplicability of Schwartz’s formula to adults, as well as the unsuitability of CKD-EPI equation for children (Pierrat et al., 2003; Michels et al., 2010; Schold et al., 2011; Zachwieja et al., 2015; Bjork et al., 2021).

With regard to vancomycin-induced nephrotoxicity, highlighted in earlier studies (Rybak M. J. et al., 2009; Elyasi et al., 2012; Gelfand and Cleveland, 2013), our work revealed that creatinine clearance, formulated to decrease with time, resulted in improved model predictions. This finding suggests that prolonged therapy may lead to a reduction in renal function (Pritchard et al., 2010). However, the influence of other risk factors like concomitant treatments and extended hospitalization requires further exploration due to limited data availability. Consequently, additional research is necessary to comprehensively understand vancomycin-induced nephrotoxicity.

For model parameter estimates, PMA50 was obtained to be 43.9 weeks, consistent with the timeline of glomerular function maturation, which nearly reaches adult levels a year after birth (Rhodin et al., 2009; Iacobelli and Guignard, 2021). The substantial RSE for *γ* might be attributed to an imbalanced distribution of PMA within the <4 years group, where approximately 40% of patients exhibited PMA values between 30 and 50 weeks (data not shown), leading to numerical difficulty in estimating the steepness factor of the sigmoid maturation function (as outlined in the Methods section). Our model indicated that central volume of distribution increased by approximately 1% per year after the age of 40, a trend in line with prior research demonstrating increased volume of distribution in the elderly (Cutler et al., 1984; Guay et al., 1993). The estimated values for clearance and central volume of distribution fell within the range of values reported in earlier studies concerning Korean patients (Bae et al., 2019; Chung et al., 2013; D. J; Kim et al., 2019), and the clearance value was closely aligned with literature values for Korean neonates (Lee et al., 2021). It is noteworthy that vancomycin pharmacokinetics have been reported to be similar across Japanese, Chinese and Caucasian patient populations (Tsai et al., 2015).

Simulations demonstrated that for adults within the normal creatinine concentration range (1.0 mg/dL), certain patient groups exceeded the optimal dose limit recommended in the label (2 g/day). Nevertheless, this higher dose is deemed acceptable, considering that individualized maintenance doses of up to 4.5 g have been suggested for certain patients, including obese patients (Rybak et al., 2020). For pediatric patients, all sub-groups proposed optimal doses below the maximum dose (3 g/day) advised in recent studies (Rybak et al., 2020). In comparison with actual administered doses in the data, the optimal dose was generally lower (data not shown), a pattern reinforced by Figure 5 where concentrations for optimal doses were consistently below those for actual doses.

**FIGURE 8:**
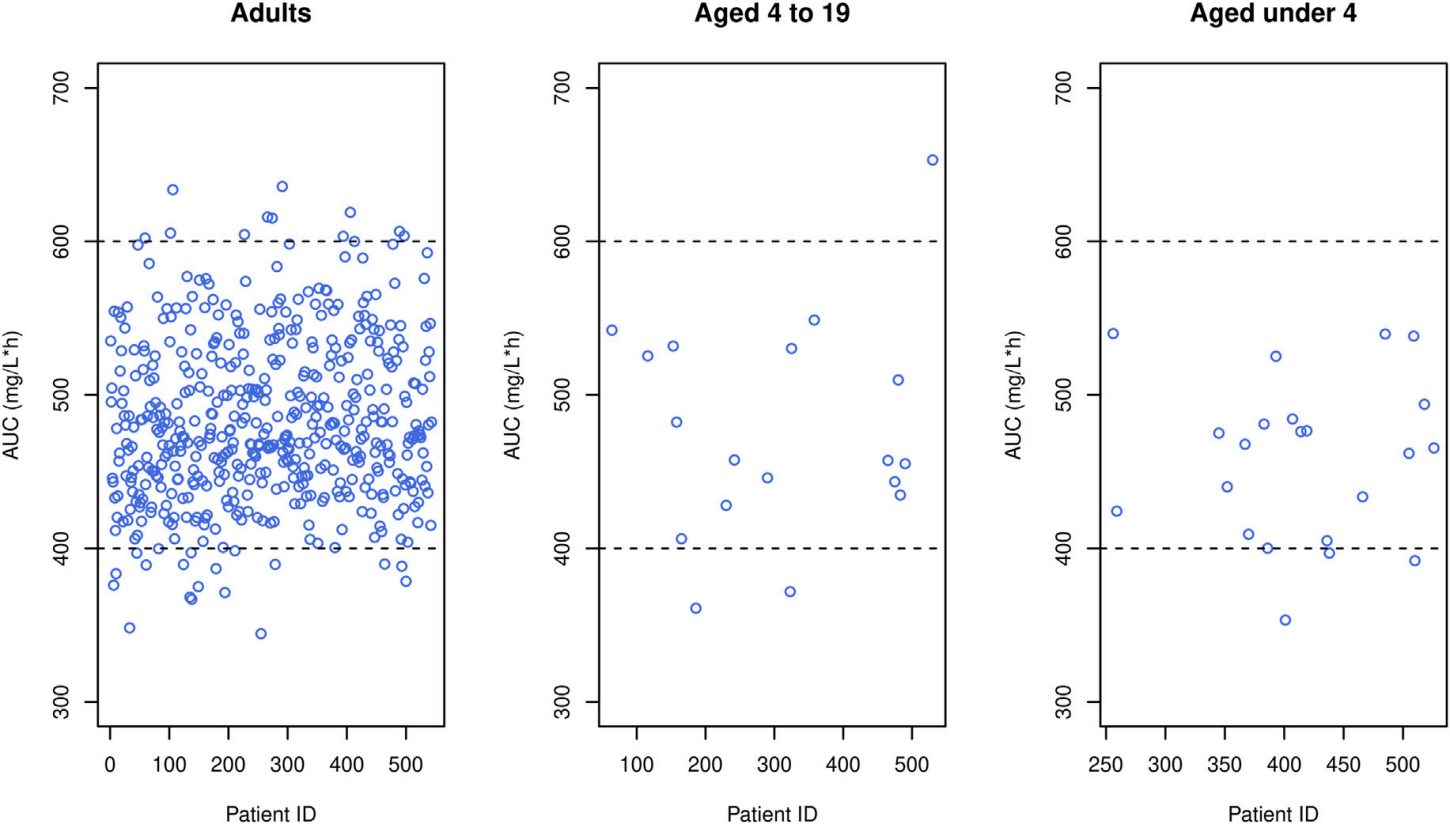
Simulated AUC (blue circles) obtained when the optimal dose was given to each patient in the data, where black horizontal dashed lines mean upper and lower limit of therapeutic range.

In our study, peak and trough concentrations were selected as indices for optimal dosage regimen design evaluation because they are conveniently available in the routine clinic, requiring only two blood samples (i.e., peak and trough samples) whereas other indices such as AUC necessitate additional blood samples and computational effort. Nevertheless, to assess the generality of our results, we simulated AUC values using the optimal doses reported in Tables 5–7 and a target AUC of 400–600 mg/L h with MIC = 1 mg/L, considering that AUC/MIC is another widely used endpoint. The resulting figure (Figure 8) indicated that around 90% of adults achieved AUC levels within the target range, and nearly all pediatric patients met the target, affirming the appropriateness of the optimal doses proposed by our study.

While other biomarkers for bacterial infection such as procalcitonin (PCT) and absolute neutrophil count (ANC) are available (Stol et al., 2019), CRP was used in our study due to its cost-effectiveness and swift test result confirmation compared to PCT and ANC, making it useful for acute infection (Escadafal et al., 2020). Despite certain limitations and potential confounders associated with CRP elevation in various conditions, its widespread use as a biomarker remains due to its practicality.

In our PD model, it was assumed that CRP proliferation increased with disease severity (D) as previously reported (Hohenthal et al., 2009; Haran et al., 2012). However, since our data lacked the requisite detail to capture the potential nonlinear relationship between CRP and D, we opted for a simple model where the production rate constant (K_in_) was multiplied by a linear function of D.

Regarding PD simulation, no discernible differences were identified in the initial response (CRP0) across different doses. This could be attributed to limitations in accurately predicting high CRP values and difficulties in isolating vancomycin’s pure effects from potential confounding factors. Nevertheless, this model exhibited trends in CRP concentration shifts based on dose and selected covariate, furnishing the optimal treatment discontinuation point at 1,100 h or 46 days. It is worth mentioning that for most adult patients, treatment was concluded around the 1100-h mark, signifying a suitable treatment duration (Figure 6).

Given the retrospective nature of this study, several limitations warrant consideration. Firstly, the information concerning medication history, including concomitant medications, was unavailable for inclusion in model building. Secondly, because laboratory tests were not performed in all patients in a clinical practice setting, CRP was only measured in 128 out of 542 patients, leading to only a limited subset of patients included in PD analysis. Lastly, the lack of follow-up data hindered the incorporation of information regarding infection recovery, relapse rates, and mortality rates.

In summation, this study established a comprehensive PK-PD model for vancomycin across all age groups and recommended optimal doses for each sub-population. Simulations underscored that most study patients’ concentrations would fall within the therapeutic range with doses smaller than usual. The R Shiny application developed herein not only facilitates identification of the optimal dose but also aids in pinpointing the optimal treatment duration by providing visual insights into simulated concentration and CRP profiles. The findings of this study are anticipated to equip clinicians with tools for achieving precise vancomycin treatment tailored to their patients.

## Data Availability

Data supporting reported results are available from the corresponding author upon approval of a written request by Severance Hospital. Requests to access these datasets should be directed to KP, kspark@yuhs.ac.
